# Protective Role of the Cholinergic Anti-Inflammatory Pathway in a Mouse Model of Viral Myocarditis

**DOI:** 10.1371/journal.pone.0112719

**Published:** 2014-11-14

**Authors:** Zheng Cheng, Ge Li-Sha, Zhao Jing-Lin, Zhang Wen-Wu, Chen Xue-Si, Chen Xing-Xing, Li Yue-Chun

**Affiliations:** 1 Department of Cardiology, Second Affiliated Hospital of Wenzhou Medical University, Wenzhou 325000, China; 2 Department of Pediatric, Second Affiliated Hospital of Wenzhou Medical University, Wenzhou 325000, China; Medical University Vienna, Center for Brain Research, Austria

## Abstract

**Background:**

Activation of the cholinergic anti-inflammatory pathway, which relies on the α7nAchR (alpha 7 nicotinic acetylcholine receptor), has been shown to decrease proinflammatory cytokines. This relieves inflammatory responses and improves the prognosis of patients with experimental sepsis, endotoxemia, ischemia/reperfusion injury, hemorrhagic shock, pancreatitis, arthritis and other inflammatory syndromes. However, whether the cholinergic anti-inflammatory pathway has an effect on acute viral myocarditis has not been investigated. Here, we studied the effects of the cholinergic anti-inflammatory pathway on acute viral myocarditis.

**Methodology/Principal Findings:**

In a coxsackievirus B3 murine myocarditis model (Balb/c), nicotine and methyllycaconitine were used to stimulate and block the cholinergic anti-inflammatory pathway, respectively. Relevant signal pathways were studied to compare their effects on myocarditis, survival rate, histopathological changes, ultrastructural changes, and cytokine levels. Nicotine treatments significantly improved survival rate, attenuated myocardial lesions, and downregulated the expression of TNF-α and IL-6. Methyllycaconitine decreased survival rate, aggravated myocardial lesions, and upregulated the expression of TNF-α and IL-6. In addition, levels of the signaling protein phosphorylated STAT3 were higher in the nicotine group and lower in the methyllycaconitine group compared with the untreated myocarditis group.

**Conclusions/Significance:**

These results show that nicotine protects mice from CVB3-induced viral myocarditis and that methyllycaconitine aggravates viral myocarditis in mice. Because nicotine is a α7nAchR agonist and methyllycaconitine is a α7nAchR antagonist, we conclude that α7nAchR activation increases the phosphorylation of STAT3, reduces the expression of TNF-α and IL-6, and, ultimately, alleviates viral myocarditis. We also conclude that blocking α7nAchR reduces the phosphorylation of STAT3, increases the expression of TNF-α and IL-6, aggravating viral myocarditis.

## Introduction

Recent studies have shown that the vagus nerve can have a positive effect on the prognosis of patients with inflammatory diseases [1]–[25]. The nervous system, via an inflammatory reflex of the vagus nerve, can inhibit cytokine release and thereby prevent tissue injury and death. This mechanism of the inflammatory reflex requires the α7nAChR, a ligand-gated ion channel expressed on macrophages, lymphocytes, neurons and other cells. When pathogens invade the body, inflammatory cytokines are produced and released by the injured tissues. These act on the solitary nucleus’ afferent sensory nerve, which in turn activates the efferent vagus nerve, promoting its terminus to release acetylcholine (Ach). The acetylcholine then stimulates α7nAchRs on the surface of inflammatory cells, hindering the biosynthesis and release of proinflammatory cytokines, which in turn inhibits local and systemic inflammatory responses. The efferent neural signaling pathway is termed the cholinergic anti-inflammatory pathway [26], and it is a neural mechanism that suppresses the innate inflammatory response [27]. The ability of the cholinergic anti-inflammatory pathway to inhibit cytokine synthesis is dependent on the α7nAchR of inflammatory cell [28]. Previous research has shown that the cholinergic anti-inflammatory pathway is involved in the regulation of inflammation in experimental sepsis, endotoxemia, ischemia/reperfusion injury, hemorrhagic shock, pancreatitis, arthritis, etc [1]–[25].

Myocarditis, which is caused by viral infection, can produce myocardial inflammation and necrosis, which can lead to heart failure, malignant arrhythmias, and even sudden cardiac death in young patients [29]. The progression of viral myocarditis can be roughly divided into three phases. In the first phase of infection, when the cardiomyocytes are attacked by the virus, viremia is followed by direct cardiomyocyte lysis. This activates the innate immune response, which involves natural killer cells, interferon-gamma, nitric oxide, etc. Antigen-presenting cells then phagocytize the released viral particles and cardiac proteins and migrate out of the heart to the regional lymph nodes. Most patients recover from this phase without significant sequelae. A subset of patients progress to the second phase of viral myocarditis, which consists of an adaptive immune response with deleterious effects on the myocardium. In this phase, T cells and antibodies are directed against viral and some cardiac epitopes, such as myosin and beta-1 receptors (“anti-heart autoantibodies”), leading to a powerful inflammatory response [30], [31], [32]. In most patients, the pathogen is eliminated, and the immune reaction is down-regulated. In others, however, the virus or inflammatory process may persist and contribute to the development of inflammatory cardiomyopathy, a form of dilated cardiomyopathy [33]. There are no specific treatment methods available for viral myocarditis, except for symptomatic treatment. Here, we studied whether the cholinergic anti-inflammatory pathway can inhibit the inflammatory process in the development of viral myocarditis.

Our study explored the effects of the cholinergic anti-inflammatory pathway on viral myocarditis and the mechanisms of those effects. α7nAcR is the core receptor of the cholinergic anti-inflammatory pathway; thus, we used nicotine, a selective α7nAchR agonist, and methyllycaconitine, a selective α7nAchR inhibitor, to stimulate and block the cholinergic anti-inflammatory pathway of mice with viral myocarditis, respectively. In these mice, we then observed survival rate, histopathologic changes, ultrastructural changes, cytokine level changes, and changes in related downstream signaling pathways.

## Materials and Methods

### Ethics Statement

The investigation conformed with the Guide for the Care and Use of Laboratory Animals published by the US National Institutes of Health (NIH Publication, 8th Edition, 2011), and all experiments were carried out in accordance with China Animal Welfare Legislation and were approved by the Wenzhou Medical College Committee on Ethics in the Care and Use of Laboratory Animals. For the survival study, individual mice were monitored daily and were euthanized when they displayed signs of myocarditis-associated morbidity such as excessive weakness and lethargy. All animals were anaesthetized with pentobarbital (100 mg/kg, one dose intraperitoneally) prior to sacrifice. Efficient anaesthesia was monitored through pinching the hind paw, when sufficiently sedated the mice were euthanized through cervical dislocation.

### Murine viral myocarditis

Specific pathogen-free inbred, 4-week-old, male Balb/C mice, obtained from the Shanghai Laboratory Animal Center, were intraperitoneally inoculated with 1.0*10^6^ plaque-forming units (pfu) of CVB3 (strain Nancy) diluted in phosphate buffered saline to a final volume of 0.1 ml. The control group was intraperitoneally inoculated with 0.1 ml normal saline solution. The day of virus inoculation was defined as day 0.

### Drug administration

Nicotine (product number: N73876) and methyllycaconitine (product number: M168) were obtained from Sigma-Aldrich Co. Twenty-four hours after the initial inoculation, nicotine (1.2 mg/kg per day, n = 40) and methyllycaconitine (2.4 mg/kg per day, n = 40) were administered by intraperitoneal injection for 14 consecutive days. Meanwhile, the control group (n = 40) and viral myocarditis group (n = 40) received the same dose of normal saline solution in the same manner. We killed 8 surviving mice from each group to extract their hearts on days 7 and 14. Every heart were divided into 3 parts. One part was used for studying myocardial histopathological and ultrastructural changes, one part for ELISA analysis of cytokine levels, the remaining part for western blot analysis of signal proteins.

### Survival analysis

Twenty mice were randomly chosen from each group for a 14-day survival analysis.

### Myocardial histopathology

After perfused with 0.9% saline solution from protrusion of left ventricle to atrium, the hearts were excised from mice. Myocardial tissue was fixed in neutral buffered formalin (10% formaldehyde in Phosphate buffered saline) over night. After fixation, placed the tissues in 70% isopropyl alcohol for 3 hours and then in each ascending strength (80%, 90%, 100% isopropyl alcohol) for 2 hours each. Then dipped the tissues in acetone for a period of 1–2 h with periodical shaking. After removing the acetone, impregnated the dehydrated tissue in paraffin wax for a period of 1 h at 58–60°C. Poured the molten paraffin into L-block along with the tissues and allowed it to become hard. Sectioned the tissue into very thin 5 µm sections using a microtome. Mounted the tissue on the slides with Mayer’s albumin solution (a mixture of equal parts of egg white and glycerin, beaten and filtered with the addition of 1% sodium salicylate) and keep in warm oven for 2 h at 60°C. Placed slides containing paraffin sections on a slide holder. Deparaffinized with Xylene for 20–30 minutes and bloted the excess xylene. Rehydrated the tissue successively with 100%, 90%, 80% isopropyl alcohol for 2–3 min. each and put it into water for 3 min. Blotted the excess water; put the tissue into Hematoxylin stain (Hematoxylin stain, Modified Harris, RICCA Chemical company Cat. No. 3530) for 1–2 min. Removed it from Hematoxylin stain and then again put it into tap water for 1–2 min. Dipped the slides containing tissue sections into 1N HCl followed by Scott’s water (Sodium Bicarbonate 3.5 g, Magnesium sulphate 20 g, distilled water 1 litre) for 1 min each. Immersed the tissue in Eosin stain (Eosin Y stain, 0.25%(w/v) in 57%(v/v) alcohol, acidified working solution, RICCA Cat. No. 2845) for 30 secs. Dehydrated the tissue successively with 80%, 90%, 100% isopropyl alcohol and finally with Xylene for 20–30 min. Placed coverslip on the slides using one drop of DPX, taking care to leave no bubbles and dry overnight to make the permanent slide. Several sections of each heart were scored blindly by two observers, and the scores for each section were averaged. The extent of cellular infiltration and myocardial necrosis were graded and scored as follows: 0 = no lesion; 1+ = lesion involving<25% of the myocardium; 2+ = lesions involving 25% to 50% of the myocardium; 3+ = lesions involving 50 to 75% of the myocardium; and 4+ = lesions involving 75% to 100% of the myocardium.

### Electron microscopy examination

The myocardial tissue was placed in cold, 6% glutaraldehyde (pH 7.3) for 1 h. The specimens were left in a refrigerator for 24 hours or longer and then washed with a phosphate buffer (pH 7.3) for 12 hours. They were then placed in cold, veronal acetate (pH 7.3) with 1 percent osmium tetroxide for 1 hour and stained with phosphotungstic acid for 10 minutes. The samples were then embedded in Ciba 502 with polymerization at 35°C for 12 hours, 45°C for 8 hours, and 60°C for 12 hours. The sections were cut with a Porter-Blum ultramicrotome. Thick (1 µm) sections were cut, stained with toluidine blue, and scanned with a light microscope to determine the areas most suitable for study. Thin sections were placed on carbon-coated 200 mesh grids. The specimens were examined with an RCA EMU-3F electron microscope at 50 KV.

### Assay of cytokine levels in myocardium

IL-6 and TNF-α levels in the myocardium were measured with an enzyme-linked immunosorbent assay (ELISA) kit manufactured by Westang Biotech Co Ltd (Shanghai, China). The sensitivity of the kits is 16 pg/ml for IL-6 and 13 pg/ml for TNF-α. The cytokine levels are expressed as pg/ml. The experiment was repeated 9 times for each sample.

### Signal pathway expression

1) Total protein extraction: The myocardial sample was cut into very small pieces using a clean razor blade and homogenized with a sonicator in RIPA buffer (RIPA buffer, Beyotime Institute of Biotechnology, Cat. NO. P0013) containing protease inhibitors (PMSF, Beyotime Institute of Biotechnology, Cat. No. ST506) and phosphatase inhibitors (Phosphatase inhibitor, Applygen Technology, Cat. No. P1260) at 4°C (the ratio among RIPA, protease inhibitor and phosphatase inhibitor was 98∶1∶1). The homogenates were centrifuged at 12,000×g for 30 min at 4°C three times, and the resulting supernatants were collected. 2) Nucleoprotein extraction: The nucleoprotein portions of the myocardial samples were extracted using NE-PER Nuclear and Cytoplasmic Extraction Reagents (NE-PER Nuclear and Cytoplasmic Extraction Reagents, Thermo scientific, Cat. No. 78833). 3) The protein concentration was determined by the Lowry method using a BCA kit (Pierce BCA Protein Assay Kit, Thermo scirntific, Cat. No. 23225). 4) Aliquots of the supernatants were diluted in an equal volume of 5× electrophoresis sample buffer (5×SDS-PAGE electrophoresis sample buffer, Beyotime Institute of Biotechnology, Cat. No. P0015) and boiled for 5 min. Protein lysates (40 µg) were separated on 12% sodium dodecyl sulfate (SDS)-poly-acrylamide electrophoresis gels and transferred onto polyvinlidene fluoride (PVDF) membranes by wet transfer technology. After being blocked with 5% non-fat dry milk in Tris-buffered saline-Tween at room temperature for 2 h, the membranes were incubated with anti-NF-kB p65 antibody(anti-NFkB p65 antibody-ChIP Grade ab7970, Abcam, at 1∶1000 dilution), anti-P-stat1 antibody (Phospho-Stat1 (Tyr701) (58D6) Rabbit mAb 9167 s, Cell Signaling Technology, at 1∶1000 dilution), anti-Stat1 antibody(Stat1 antibody 9172 s, Cell Signaling Technology, at 1∶1000 dilution), anti-P-stat3 antibody(Phospho-Stat3 (Tyr705) (D3Α7) XP Rabbit 9145 s, Cell Signaling Technology, at 1∶1000 dilution), and anti-Stat3 antibody (Stat3 (124H6) Mouse mAb 9139 s, Cell Signaling Technology, at 1∶1000 dilution) at 4°C overnight. Then, the membranes were incubated with horseradish peroxidase-conjugated second antibody (NFkB p65, P-stat1, stat1, P-stat3 with Goat polyclonal Secondary Antibody to Rabbit IgG - H&L (HRP) (ab6721) at 1∶20000 dilution; stat3 with Goat polyclonal Secondary Antibody to Mouse IgG1 - heavychain (HRP) (ab97240) at 1∶2000 dilution) at 4°C overnight. The blots were visualized with Western blotting luminol reagent (sc-2048, Santa Cruz Biotechnology, CA, USA) using the Electrophoresis Gel Imaging Analysis System (MFChemiBIS3.2, DNR Bio-Imaging Systems, ISR). Subsequently, densitometric analyses of the bands were semi-quantitatively conducted using Scion Image Software (Scion Corporation, Maryland, USA). The relative protein levels were calculated by comparison with the levels of the loading control, GAPDH (# G13–61 M, Signalchem, Canada). The experiment was repeated 9 times for each sample.

### Statistical Analysis

All values were expressed as Mean ± Standard deviation (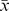
±s). Survival rate was analyzed by the Kaplan-Meier method. The statistical analysis was performed using a one-way analysis of variance (ANOVA), followed by Fisher’s protected least significant difference test. The correlation analysis was performed using Pearson Correlation. Analysis was performed with SPSS 17.0 statistical software. A P value of <0.05 was considered as statistically significant

## Results

### Survival Rate

**Figure 1:**
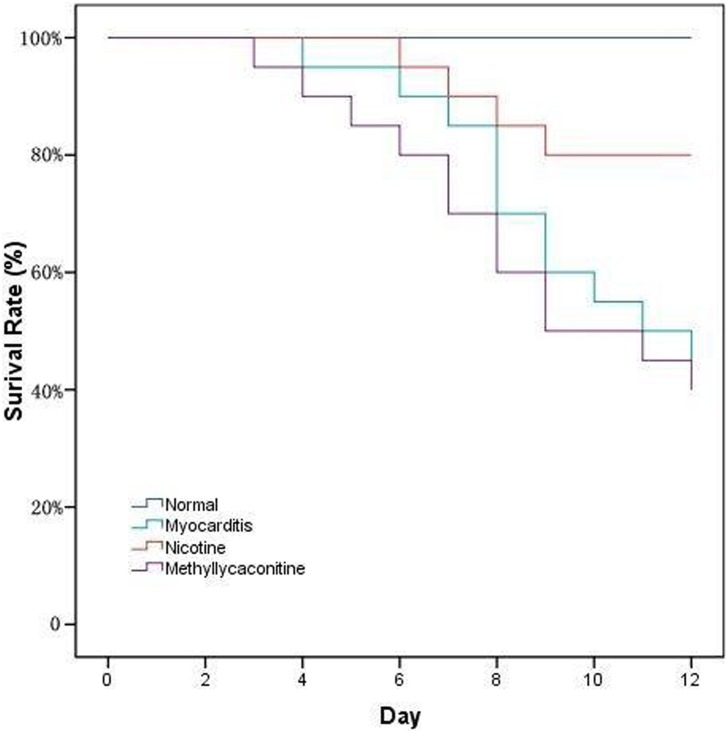
The effects of nicotine and methyllycaconitine on survival rate. The day of virus inoculation was defined as day 0, during the 14-day observation, there were no deaths happened in the control group. In the other three groups, the period from day 5^th^ to 10^th^ was demonstrated to be the death peak, the survival rate of the CVB3-inoculated was 45% for those treated with saline, 80% for those treated with nicotine, and 40.0% for those treated with methyllycaconitine. Compared to the myocarditis and methyllycaconitine groups, the survival rate was significantly higher in the nicotine group (P<0.05).

There were no deaths in the control group during the 14-day observation period. The survival rate of the CVB3-inoculated mice after 14 days was 45% for those treated with saline, 80% for those treated with nicotine, and 40.0% for those treated with methyllycaconitine. The day of virus inoculation was defined as day 0, and the period from day 5^th^ to 10^th^ was demonstrated to be the death peak. Compared to the myocarditis and methyllycaconitine groups, the survival rate was significantly higher in the nicotine group (Figure 1).

### Myocardial Histopathology

**Figure 2:**
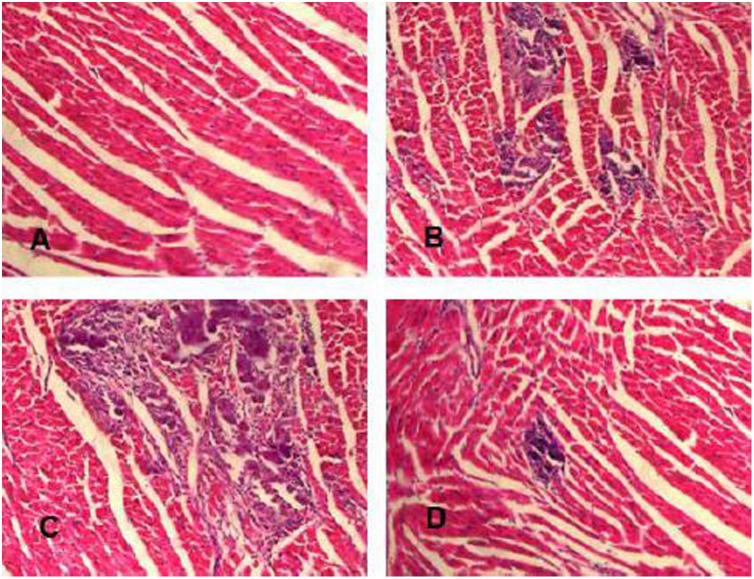
Myocardial histopathology (Hematoxylin Eosin×200) in the heart. (A) Histopathology of control group (grade 0). (B) Representative histopathology of myocarditis group. The extent of lesions was less severe in the myocarditis group than in the methyllycaconitine group; several small foci of cellular infiltrations in the inflammatory region are shown (grade 2). (C) Representative histopathology of methyllycaconitine group. The extent of lesions was the most severe; large foci of cellular infiltrations in the inflammatory region were observed (grade 3). (D) Representative histopathology of nicotine group. The area of cellular infiltrations was much smaller and limited in the inflammatory region than in the myocarditis group and methyllycaconitine group (grade 1).

The mice in the myocarditis group sacrificed on days 7 and 14 showed severe injuries to the myocardium with cellular infiltration and necrosis. The severity of cellular infiltration and necrosis was lower in the nicotine group and higher in the methyllycaconitine group compared with the myocarditis group (Figure 2, Table 1).

**Table 1:** Myocardial pathological score of the four groups.

		Infiltration		Necrosis	
		7 d	14 d	7 d	14 d
Normal	8	ND	ND	ND	ND
Nicotine	8	1.2±0.2*	0.7±0.1*	1.0±0.1*	1.2±0.1*
Myocarditis	8	1.9±0.2	1.0±0.1	1.4±0.1	1.5±0.1
Methyllycaconitine	8	2.8±0.2*	1.8*±0.1*	1.8±0.2*	2.1±0.1*

ND, not detected.

*P<0.05, versus Myocarditis.

### Myocardial Ultrastructural Changes

**Figure 3:**
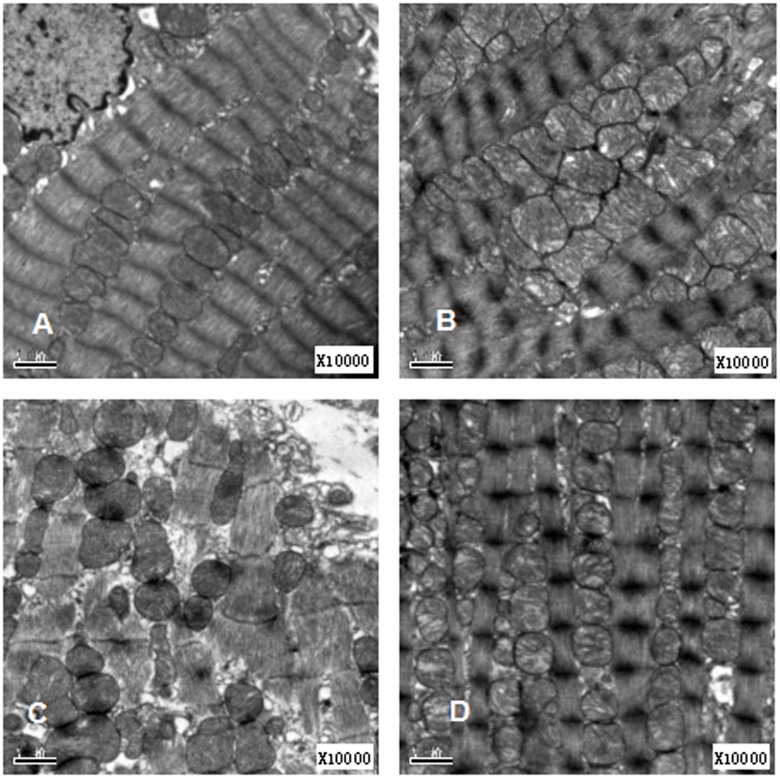
Electron microscope changes at day 7 (×10000). (A) The normal control group. (B) The myocarditis group. (C) The methyllycaconitine treatment group (D) The nicotine treatment group.

Under the electron microscope, the ultrastructure of the myocarditis, nicotine, and methyllycaconitine groups was markedly different from that of the control group (Figure 3). For normal control group, the myocardial cells had integrated membranes. The cytoplasm of the myocardial cells at the longitudinal section was full of aligned myofibrils; the myofibrils were divided neatly into sarcomeres by the Z line. We also observed abundant mitochondria in the cytoplasm; most mitochondria had a round or oval shape and were located between myofibrils with intensive cristae in the inner mitochondrial space (Figure 3A). In the myocarditis group, we observed a breakdown of a portion of the myofibrils; the Z lines were obscure, and the mitochondria become swollen, with few cristae (Figure 3B). Compared with the myocarditis group, the ultrastructural changes of the methyllycaconitine treatment group were much more severe: the myofibrils were completely destroyed; the sarcomere structures were nearly unidentifiable; only a few mitochondria were present; and the remaining mitochondria were severely swollen. Mitochondrial cristae were often widely separated, and small intramitochondrial vesicles were observed (Figure 3C). Compared with the myocarditis group, the ultrastructural changes of the nicotine treatment group were less severe. In the nicotine group, the myofibrils became thinner and disorganized, the Z lines became slightly obscured, the sarcomeres remained integral, and the mitochondria were slightly swollen (Figure 3D).

### ELISA analysis of cytokine levels in myocardium

**Figure 4:**
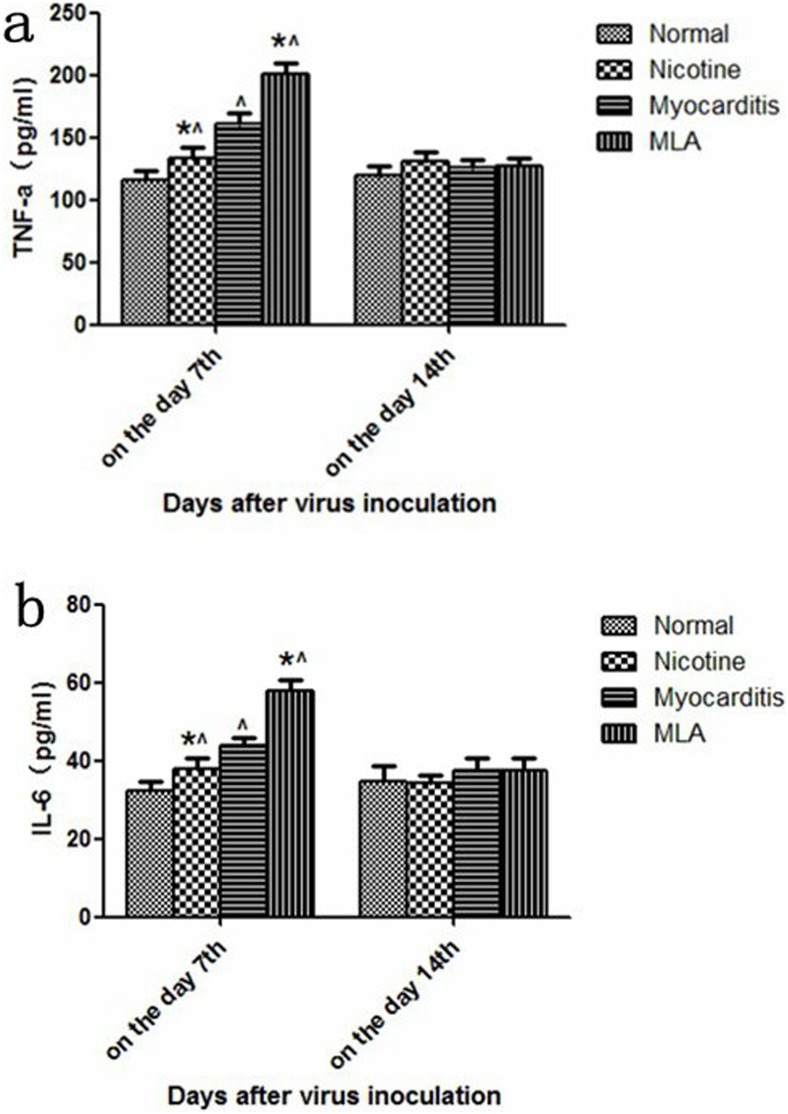
TNF-α and IL-6 levels in the myocardial tissues of mice on days 7 and 14. *P<0.05, versus myocarditis group; ∧P<0.05, versus control group. MLA, methyllycaconitine.

On day 7, compared to the myocarditis group, the levels of TNF-α and IL-6 were significantly lower in the nicotine group (TNF-α: Nicotine 133.75±8.42 pg/ml vs Myocarditis 160.60±8.75 pg/ml; IL-6: Nicotine 38.00±2.80 pg/ml vs Myocarditis 44.00±1.82 pg/ml, P<0.05) (Figure 4) and were higher in the methyllycaconitine group (TNF-α: Methyllycaconitine 200.25±8.73 pg/ml vs Myocarditis 160.60±8.75 pg/ml; IL-6: Methyllycaconitine 58.02±2.56 pg/ml vs Myocarditis 44.00±1.82 pg/ml, P<0.05) (Figure 4). On day 14, no obvious differences in the levels of IL-6 and TNF-α were observed between the nicotine group, methyllycaconitine group and myocarditis group (TNF-α.: Nicotine 130.50±8.20 pg/ml vs Methyllycaconitine 127.50±5.50 pg/ml vs Myocarditis 125.25±6.55 pg/ml, P>0.05, IL-6: Nicotine 34.25±2.20 pg/ml vs Methyllycaconitine 37.65±2.90 pg/ml vs Myocarditis 37.50±3.10 pg/ml, P>0.05).

### Western blot of signal protein in myocardium

**Figure 5:**
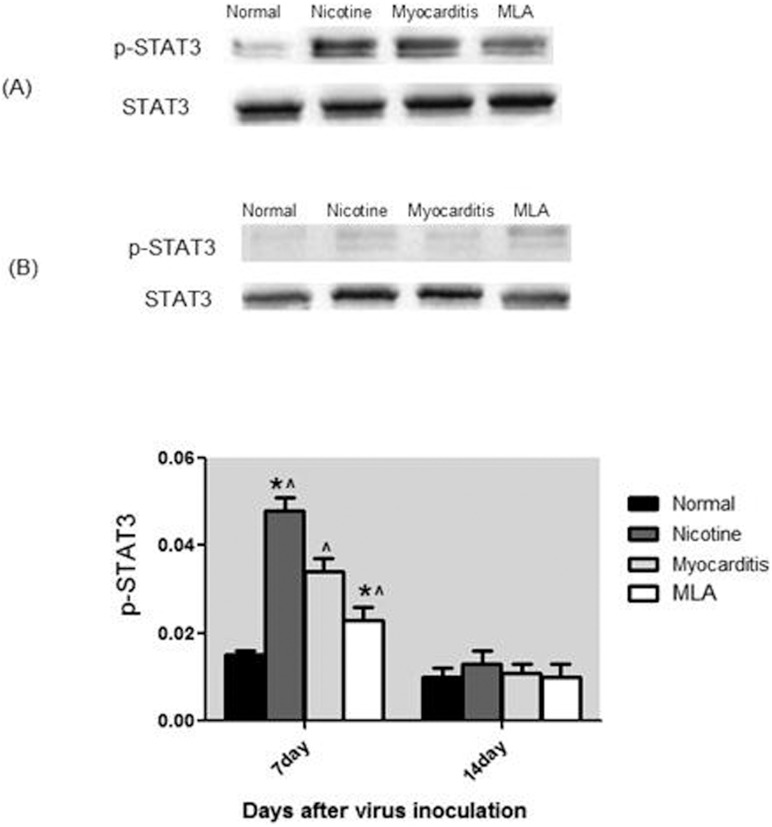
Expression of p-STAT3 in the four groups. (A) p-STAT3 level of four groups on day 7. (B) p-STAT3 level of four groups on day 14. *P<0.05, versus myocarditis group; ∧P<0.05, versus control group. MLA, methyllycaconitine.

**1) p-STAT3/STAT3:** On day 7, compared with the control group, the p-STAT3 levels of the nicotine, myocarditis, and methyllycaconitine groups were upregulated. The p-STAT3 level was highest in the nicotine group, followed by the myocarditis group and then the methyllycaconitine group; there were significant differences in the p-STAT3 levels among these four groups (p-STAT3: Nicotine 0.048±0.003 vs Myocarditis 0.034±0.003 vs Methyllycaconitine 0.023±0.003 vs Normal 0.015±0.001, P<0.05) (Figure 5). On day 14, no significant differences in the p-STAT3 level was found among the four groups (p-STAT3: Nicotine 0.013±0.003 vs Myocarditis 0.011±0.002 vs Methyllycaconitine 0.010±0.003 vs Normal 0.010±0.002, p>0.05) (Figure 5).

**Figure 6:**
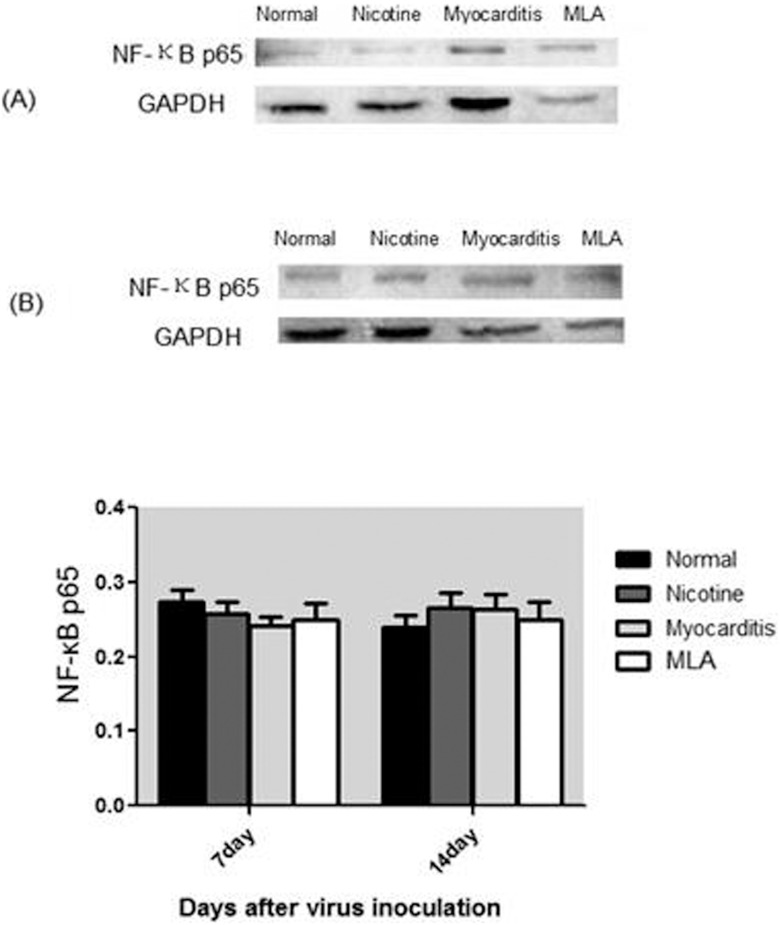
Expression of NF-κB p65 in the four groups. (A) NF-κB p65 level of four groups on day 7; (B) NF-κB p65 level of four groups on day 14. MLA, methyllycaconitine.

**2) p-STAT1/STAT1:** The expression of p-STAT1 was hardly detectible. Thus, we could not determine whether p-STAT1 was a downstream effector of α7nAchR.
**3) NF-κB p65 in cellular nucleus:** On day 7 and 14, the level of NF-κB p65 in the cellular nuclei of the four groups was not significantly different (on day 7, NF-κB p65: Nicotine 0.257±0.017 vs Myocarditis 0.253±0.011 vs Methyllycaconitine 0.256±0.021 vs Normal 0.273±0.017, P>0.05; on day 14, NF-κB p65: Nicotine 0.266±0.019 vs Myocarditis 0.264±0.019 vs Methyllycaconitine 0.250±0.023 vs Normal 0.240±0.015, P>0.05) (Figure 6).

### The correlation between p-STAT3, NF-κB and cytokines

**Figure 7:**
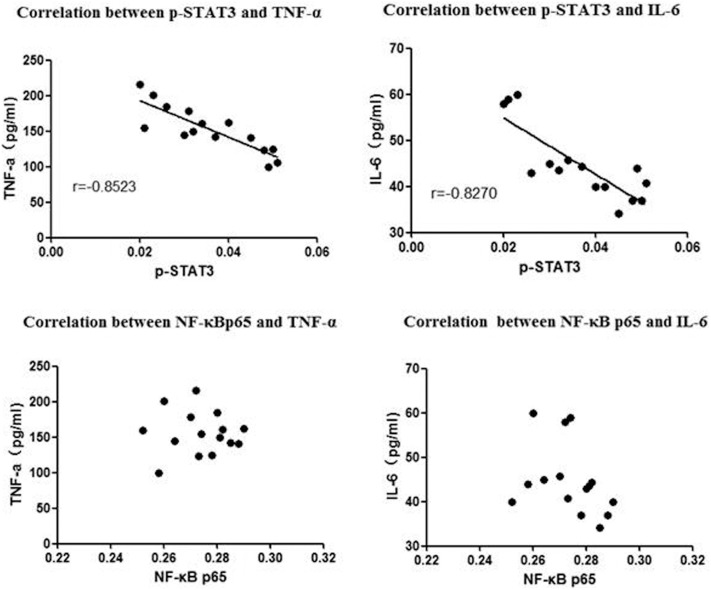
Correlation between p-STAT3, NF-κB and cytokines (TNF-α and IL-6 ) **on day 7.** The bivariate correlation analysis fit a straight line to the significant negative relationship between the expression of p-STAT3 and the levels of TNF-α and IL-6 on day 7 (upper panel). However, the levels of TNF-α and IL-6 were not correlated with the expression of NF-κB p65 (lower panel).

We used the Pearson’s correlation analysis method to assess the relationship between the expression of p-STAT3 and NF-κB and the levels of the inflammatory cytokines TNF-α and IL-6. The bivariate correlation analysis fit a straight line to the significant negative relationship between the expression of p-STAT3 and the levels of TNF-α (r = −0.8523; Figure 7) and IL-6 (r = −0.8270; Figure 7) on day 7. However, the levels of TNF-α and IL-6 were not correlated with the expression of NF-κB p65 (Figure 7).

## Discussion

Activation of the cholinergic anti-inflammatory pathway has been sufficiently proven to relieve inflammatory responses and improve the prognosis of patients with experimental sepsis, endotoxemia, ischemia/reperfusion injury, hemorrhagic shock, pancreatitis, arthritis and other inflammatory syndromes. With that in mind, Hong Li hypothesized that cholinergic anti-inflammatory pathway stimulation can be used to treat myocarditis and can relieve symptoms and inhibit inflammation [34]. Christoph Leib reported that the activation of the cholinergic anti-inflammatory pathway with nicotine reduces inflammation in patients with autoimmune myocarditis [14]. However, whether the cholinergic anti-inflammatory pathway plays part in viral myocarditis has never been studied. Our study aimed to explore the effect of the cholinergic anti-inflammatory pathway on viral myocarditis. To the best of our knowledge, this is the first study to investigate the effects of the cholinergic anti-inflammatory pathway in acute viral myocarditis.

We studied the survival rate, myocardial histopathology, ultrastructural changes, and cytokine levels in the four groups and found that treatment with nicotine in CVB3-infected mice improved the survival rate, relieved the pathological and ultrastructural lesions and markedly suppressed the expression of TNF-α and IL-6. Furthermore, treatment with methyllycaconitine caused the opposite effects. This study revealed for the first time that acute viral myocarditis is significantly ameliorated by treatment with the α7nAchR agonist nicotine and is aggravated by treatment with the selective α7nAchR antagonist methyllycaconitine. By comparing nicotine and methyllycaconitine’s effects on viral myocarditis, we concluded that the inflammatory aspects of viral myocarditis were alleviated by stimulating and aggravated by blocking the cholinergic anti-inflammatory pathway.

It is well known that stimulation of a7nAChR by nicotine or acetylcholine initiates the cholinergic anti-inflammatory pathway. Nicotine, a nicotinic cholinergic agonist, could bind to α7nAChR and then activate cholinergic anti-inflammatory pathway. Wang H et al in 2003 firstly demonstrate that α7nAChR subunit played a key role in supressing cytokine production in response to nicotine stimulation. After treating the wild type mice and α7nAChR knockout mice with bacterial endotoxin of lipopolysaccharide (LPS), in macrophages derived from wild type mice, nicotine exerted decrease of pro-inflammatory cytokines, however, macrophages derived from α7nAChR knockout mice were refractory to nicotine, and produced pro-inflammatory cytokines normally [28]. Animal study also indicated that nicotine treatment could down-regulate the production of IL-6 and TNF-α, the recruitment of leukocytes after LPS stimulation [15]. For the effect of nicotine during virus or virus-like infection, it was reported that poly (I:C)-induced inflammatory response in mouse macrophages could be suppressed by nicotine significantly. Specifically, nicotine could attenuate the mRNA expression and production of IL-6 and TNF-α in poly (I:C) stimulated peritoneal macrophages [16]. Nicotine pretreatment might prevent the mice from renal dysfunction during kidney ischemia/reperfusion injury by binding to the α7nAChR, and then preventing neutrophil recruitment, decreasing tubular damage as well as reducing the production of TNF-α [17]. As for methyllycaconitine, many studies chosed it as a selective α7nAChR antagonist [13], [25]. In our study, nicotine and methyllycaconitine were selected to stimulate and block the cholinergic anti-inflammatory pathway. In agreement with previous studies, we found that nicotine reduced the expression of TNF-α and IL-6, and methyllycaconitine increased the expression of TNF-α and IL-6. Therefore, α7nAChR may be mainly targeted by nicotine and methyllycaconitine in the effects of cholinergic anti-inflammatory pathway on viral myocarditis.

In our study, western blot analysis of molecules of related signaling pathways was performed to clarify how the cholinergic anti-inflammatory pathway functions in viral myocarditis. Previous studies indicated that the combination of acetylcholine and the α7nAChR can inhibit the NF-KB transcription factor and activate the Jak2-STAT3 signaling pathway. De Jonge et al. reported that stimulation of the vagus nerve ameliorated surgery-induced inflammation and postoperative ileus by activating STAT3 [2]. They concluded that the vagal anti-inflammatory pathway is activated by alphα7 subunit-mediated Jak2-STAT3 activation. STAT3 was phosphorylated by the tyrosine kinase Jak2, which is recruited to the α7 subunit of the nicotinic acetylcholine receptor. The anti-inflammatory effect of nicotine is dependent on the ability of phosphorylated STAT3 to bind and transactivate its DNA response elements and, consequently reduce the transcription of cytokines [35]. Altavilla et al. reported that activation of the cholinergic anti-inflammatory pathway reduced NF-κB activation, blunted TNF-α production, and relieved the inflammatory response [3]. Related studies have shown that when NF-κB p65 combines with NF-κB p50 to form a dimer, that dimer inhibits IκB by retaining it in the cytoplasm where is has no function. When IκB kinase (IKK), an upstream molecule of IκB, is phosphorylated, it can degrade IκB. Without the constraint of IκB, NF-κB p50 will transport NF-κB p65 to the cell nucleus. NF-κB p65 binds to its related DNA response elements, which increases the transcription of TNF-α and, consequently, aggravates the inflammatory response [36], [37], [38].

We studied p-STAT1, p-STAT3, and NF-κB p65. The expression of p-STAT1 was undetectable; thus, we cannot ascertain whether it is a downstream effector of α7nAchR. Compared to the control group, the levels of p-STAT3 were increased in the nicotine, myocarditis and methyllycaconitine group on day 7, and were highest in the nicotine group, followed by the myocarditis group and the methyllycaconitine group. The Person correlation analysis showed that on day 7, the TNF-α and IL-6 levels in the myocarditis, nicotine and methyllycaconitine groups were negatively correlated with their corresponding p-STAT3 levels. Thus, we inferred that in mice with viral myocarditis, nicotine activated α7nAchR, increasing the phosphorylation STAT3; this resulted in the down-regulation of the expression of the cytokines TNF-α and IL-6 and relieved the inflammatory response. We also inferred that in mice with viral myocarditis, methyllycaconitine blocked α7nAchR, decreasing the phosphorylation of STAT3; this resulted in the up-regulation of the expression of the cytokines TNF-α and IL-6 and aggravated the inflammatory response. In our study, the levels of NF-κB p65 in the cellular nuclei were not significantly different among of the four groups, and there were no linear relationships between NF-κB p65 and TNF-α or IL-6 levels; thus we could not determine whether NF-κB p65 takes part in the cholinergic anti-inflammatory pathway.

The results of our study show that stimulating cholinergic anti-inflammatory pathway plays a protective role in viral myocarditis. Its mechanism is related to the activity of p-STAT3 after α7nAChR is activated. When nicotine acts on inflammatory cells via the recruitment of Jak2 to the α7nAChR and the activation of Jak2, the anti-inflammatory molecule STAT3 is activated, and when methyllycaconitine acts on inflammatory cells, the reverse occurs. STAT3 is a negative regulator of the inflammatory response; previous studies have shown the activation of the STAT3 cascade after α7nAChR ligation is consistent with the observed inhibition of proinflammatory cytokines. We have shown that the recruitment of inflammatory infiltrates induced by viral myocarditis were reduced substantially by stimulation of the cholinergic anti-inflammatory pathway and increased substantially by stimulation of the cholinergic anti-inflammatory pathway. However, Nicolussi et al. [39] found that the cholinergic anti-inflammatory pathway efficiently counteracts T cell infiltration into the neurodegenerative central nervous system, but cannot counteract central nervous system inflammation in experimental autoimmune encephalomyelitis. The results look contradictory and are not in agreement with previous and present studies [1], [3]–[28]. The discrepancy between the effects of cholinergic anti-inflammatory pathway on many inflammatory diseases might relate to the different pathophysiological mechanisms in these different models, the determined tissue, the treatment duration, or animal species. Tissue injury in the experimental autoimmune encephalomyelitis model reported by Nicolussi et al may be not only driven by the action of cytokines, but also by demyelinating antibodies induced by their immunization protocol [39].

## Conclusions

In acute viral myocarditis, activation of α7nAchR increases the phosphorylation of STAT3, reduces the levels of TNF-α and IL-6, and, ultimately, alleviates viral myocarditis. Moreover, the inhibition of α7nAchR reduces the phosphorylation of STAT3, increases the levels of TNF-α and IL-6, and, ultimately, aggravates viral myocarditis. The results of our study may aid the development of therapeutic strategies for viral myocarditis.
